# Divergent Regulation of OCT and MATE Drug Transporters by Cadmium Exposure

**DOI:** 10.3390/pharmaceutics13040537

**Published:** 2021-04-13

**Authors:** Hong Yang, Shiwei Zhou, Dong Guo, Obinna N. Obianom, Qing Li, Yan Shu

**Affiliations:** 1 Department of Clinical Pharmacology, Xiangya Hospital, Central South University, Changsha 410008, China; hong.yang@umaryland.edu (H.Y.); zhoushiwei@csu.edu.cn (S.Z.); 2 Department of Pharmaceutical Sciences, School of Pharmacy, University of Maryland at Baltimore, Baltimore, MD 21201, USA; dguo@rx.umaryland.edu (D.G.); oobianom@umaryland.edu (O.N.O.); 3 Department of Thyroid Surgery, The Second Xiangya Hospital, Central South University, Changsha 410011, China

**Keywords:** cadmium, organic cation transporters (OCTs), multidrug and toxin extrusion proteins (MATEs), liver, kidney, candesartan

## Abstract

Coordinated transcellular transport by the uptake via organic cation transporters (OCTs) in concert with the efflux via multidrug and toxin extrusion proteins (MATEs) is an essential system for hepatic and renal drug disposition. Despite their clinical importance, the regulation of OCTs and MATEs remains poorly characterized. It has been reported that cadmium (Cd^2+^) increase the activities of OCTs while being a substrate of MATEs. Here, we found that human (h) OCT2 protein, as compared with hMATE1, was more active in trafficking between the plasma membrane and cytoplasmic storage pool. Cd^2+^ exposure could significantly enhance the translocation of hOCT2 and hOCT1, but not hMATE1, to the plasma membrane. We further identified that candesartan, a widely prescribed angiotensin II receptor blocker, behaved similarly toward OCT2 and MATE1 as Cd^2+^ did. Importantly, Cd^2+^ and candesartan treatments could lead to an enhanced accumulation of metformin, which is a well-characterized substrate of OCTs/MATEs, in mouse kidney and liver, respectively. Altogether, our studies have uncovered possible divergent regulation of OCTs and MATEs by certain xenobiotics, such as Cd^2+^ and candesartan due to the different cellular trafficking of these two families of transporter proteins, which might significantly affect drug disposition in the liver and kidney.

## 1. Introduction

The human genome encodes more than 400 membrane transporters that function as gatekeepers in determining the selective permeability of the cell membrane [1]. Notably, the hepatocytes and the proximal tubular cells in the renal cortex are highly equipped with a variety of membrane transporters that are essential for hepatic and tubular uptake and the secretion of numerous endogenous and exogenous chemicals, including therapeutic drugs [2,3]. For instance, nearly one-third of the top 200 prescribed drugs in the U.S. (2010) are predominantly eliminated via urine [4], many of which undergo net renal secretion mediated by the membrane transporters expressed in the kidney [2,5].

The organic cation transporters and multidrug and toxin extrusion proteins (OCTs/MATEs) are pairs of the solute carrier (SLC) transporter families highly expressed in the basolateral and apical membrane of hepatocytes (OCT1 and MATE1 in human and mouse) and renal proximal tubular cells (OCT2 and MATE1/2-K in human, OCT1/2 and MATE1 in mouse), respectively [6]. Due to the large overlap in their substrates, OCTs and MATEs often function in tandem to vectorially transport chemicals from blood into bile or urine. Many endogenous and exogenous chemicals have been identified as their substrates, such as creatinine, metformin, and cisplatin. The distribution, elimination, therapeutic response, and toxicity of a clinical drug can be mainly determined by the activities of OCTs/MATEs [7,8,9,10,11]. Hence, it is recommended by the US FDA for the pharmaceutical industry to examine the potential of their new molecular entities (NMEs) as substrates or inhibitors of OCTs/MATEs in order to avoid drug–drug interaction mediated by these transporters [1,12].

Despite their crucial importance in drug disposition and response, the regulation of OCTs/MATEs is still poorly characterized. Protein kinase A (PKA), protein kinase C (PKC), Yes1 kinase, phosphatidylinositol 3-kinase (PI3K)- and calmodulin-dependent kinases have been reported to regulate the function of OCT2 at the post-translational level [13,14,15,16,17]. Recently, we have demonstrated that OCT2 membrane translocation involves protein–protein interaction between serine/threonine-protein kinase AKT2, calcium/calmodulin, and the AKT substrate AS160 [18]. However, the detailed molecular mechanism remains to be depicted, and the regulation has yet to be verified in vivo. In addition, the role of protein trafficking, which is a process that is vital in the membrane expression and activity of transporters [19,20], in the regulation of OCTs and MATEs needs to be further examined.

Previously, the heavy metal cadmium (Cd^2+^) has been reported to be a substrate of OCTs [21,22]. We have also demonstrated Cd^2+^ as a substrate of MATEs [23,24], and the ion can increase the activity of OCT2 recently [18]. Although it has no physiological benefit, Cd^2+^ exposure can alter multiple molecular signaling pathways [25], some of which have been indicated in regulation of OCTs and MATEs as described above. These findings have suggested a potential Cd^2+^–drug interaction mediated by OCTs/MATEs. Thus, we hypothesized that Cd^2+^ exposure would have a regulatory effect on the activity of drug transporters such as OCTs/MATEs, leading to variation in drug disposition and response.

In this study, we firstly confirmed that Cd^2+^ exposure led to an increased activity of OCTs as reported recently [18], while possessing an inhibitory effect on MATEs in HEK293 cells. Then, the mechanism underlying these divergent effects of Cd^2+^ exposure on transporter activities was explored. Moreover, we examined the effects of Cd^2+^ exposure on the pharmacokinetics and tissue accumulation of the OCT/MATE substrate metformin in mice. In addition, with a small scale of screening, we identified that candesartan, a clinically used drug, behaved similarly toward OCT2/MATE1 as Cd^2+^ did. Our results indicated that drug disposition could be affected by certain xenobiotics, such as Cd^2+^ and candesartan, through their divergent regulation on the activities of OCTs and MATEs.

## 2. Materials and Methods

### 2.1. Antibodies and Reagents

Primary antibodies were from Cell Signaling (Danvers, MA, USA): Na, K-ATPase (#3010, polyclonal); Santa Cruz Biotech (Dallas, TX, USA) pan-Cadherin (sc-515872, monoclonal), MATE1 (sc-133390, polyclonal); Thermo Fisher Scientific (Waltham, MA, USA): SLC22A2 (PA5-37290, polyclonal), SLC22A1 (MA5-15730, monoclonal); Sigma (St. Louis, MO, USA): beta-Actin (A3854, monoclonal). Reagents were from Sigma: cadmium chloride (202908), Filipin (F4767), MG-132 (474790), cycloheximide (C1988); EMD Milipore (Burlington, MA, USA): cisplatin (232120); Biolegend (San Diego, CA, USA): brefeldin A (420601); Moravek (Brea, CA, USA): [^14^C]-labeled metformin hydrochloride (MC2043); PerkinElmer (Waltham, MA, USA): [^3^H]-labeled MPP+ (NET914250UC), [^14^C]-labeled tetraethylammonium bromide (NEC298250UC); Thermo Fisher Scientific: streptavidin agarose (20349), sulfo-NHS-SS-biotin (21217). Trace grade reagents were used for metal quantification on inductively coupled plasma–mass spectrometry (ICP-MS, Agilent Technologies, Santa Clara, CA, USA). All other reagents were commercially available.

### 2.2. Cell Culture and Transfection

HEK-293 cell lines stably expressing human OCT2 (HEK-hOCT2), human MATE1 (HEK-hMATE1), human MATE2-K (HEK-hMATE2-K), and human OCT1 (HEK-hOCT1) were generated by using the Flp-In system (Invitrogen) as described previously [26,27]. The cells were cultured in DMEM supplemented with 10% FBS, streptomycin (100 µg/mL), and penicillin (100 U/mL), and maintained in at 37 °C in a humidified atmosphere with 5% CO_2_. Transient overexpression of mOCT1, mOCT2, and mMATE1 were achieved with either lipofectamine 2000 or a calcium-based transfection, which has been described elsewhere [26,28].

### 2.3. Mouse Primary Renal Tubular Cell Isolation and Culture

Mouse primary renal tubular cells were isolated and cultured according to published protocols with minor modification [29,30]. In brief, dissected mouse renal cortex were minced and digested with collagenase II. Mouse tubulars were enriched and filtered through sieves with pore sizes of 300 µm and then 40 µm. Renal tubular cells were cultured in collagen-coated plated with advanced DMEM/F12 media for 3 to 5 days to reach 50 to 80% confluence.

### 2.4. Cell Surface Biotinylation

Membrane proteins on the cell surface were labeled with membrane-impermeable biotinylation reagent, sulfo-NHS-SS-biotin, and pulled down by the streptavidin agarose beads as described previously [20]. In brief, cells in a 24-well plate were incubated with 0.3 mL of sulfo-NHS-SS-biotin (1 mg/mL) in calcium and magnesium-enriched phosphate-buffered saline (PBS-CM) (137 mM NaCl, 2.7 mM KCl, 4.3 mM Na2HPO4, 1.4 mM KH2PO4, 0.1 mM CaCl2, and 1 mM MgCl2, pH 7.3) for 1 h at 4 °C with gentle shaking. Sulfo-NHS-SS-biotin was completely quenched by adding 0.5 mL Tris buffer (1.0 mM, pH = 8.0), and was briefly rinsed with ice-cold PBS-CM. Then, the cells were lysed with lysis buffer (1% NP-40 with protease inhibitor mixture, 100 µL). Then, streptavidin–agarose beads (50 µL) was added to 100 µg of the crude membrane proteins, and it was incubated on a rocker at 4 °C overnight. Membrane proteins were enriched by incubating the beads with urea sample buffer (10 mM Tris, 8M urea, 10% glycerol, 1% SDS and 1/20 beta-mercaptoethanol, 100 µL) for 1 h, after being washed for 3 times with the same lysis buffer.

### 2.5. Immunoblotting Analysis

Samples from cell lysate and tissues were analyzed by immunoblotting (Western blot). To avoid protein aggregation [31], all membrane proteins were prepared in the urea sample buffer as described above and incubated for 30 min at room temperature before electrophoresis, while a standard boiling of 10 min in Laemmli sample buffer was used to prepare all other proteins samples. All proteins were transferred into a nitrocellulose membrane (0.45 µm). Membranes were blocked in 5% milk to reduce unspecific binding. After incubation with primary antibodies overnight at 4 °C, membranes were incubated with HRP-conjugated second antibodies at room temperature for 1 h, then visualized by adding Western Lightning Plus-ECL (PerkinElmer, Waltham, MA, USA). The images were captured by Odyssey Fc imaging system (Li-cor, Lincoln, NE, USA).

### 2.6. Analysis of Protein Degradation Half-Life

HEK-hOCT2 and HEK-hMATE1 cells were incubated with cycloheximide (CHX, 50 µg/mL) to inhibit protein synthesis. The proteasome inhibitor MG-132 (10 µM) was added along with CHX as control to inhibit protein degradation. Following incubation for varied times (0 to 10 h for HEK-hOCT2, 0 to 32 h for HEK-hMATE1), cells were harvested and lysed, and cell lysates were prepared as described above. Protein abundance was analyzed by Western blotting as described above. Desired protein bands were quantified using the Image Studio Lite 5.2.5 (Licor). Beta-actin was used as the loading control, as CHX treatment did not affect its level as previously reported [32]. Protein degradation rate is expressed as half-life (t_1⁄2_), which is the time for degradation of 50% of the protein. Each of the half-life values reported was calculated from three independent determinations and is expressed as mean ± S.E.M.

### 2.7. Cellular Uptake Assay and Cd Exposure

Metformin (50 µM, with 1/5 of [^14^C]-metformin), [^14^C]-TEA (10 µM), MPP^+^ (10 µM, with 1/1000 of [^3^H]-MPP^+^), and cisplatin (100 µM) was used in the cellular uptake studies in mouse primary renal tubular cells and the cells stably or transiently expressing hOCT1, hOCT2, hMATE1, hMATE2-K, mOCT1, mOCT2, or mMATE1. The uptake time was set according to the literature [7,33,34,35,36]. In brief, the uptake of metformin, TEA, MPP^+^ and cisplatin in hOCT2 were set to 20 s, 5 min, 2 min, and 30 min respectively; the uptake of metformin in hOCT1, mOCT1, mOCT2, hMATE1, hMATE2-K, and mMATE1 were set to 10, 1, 1, 2, 2, and 2 min, respectively. The uptake was performed in Krebs-Ringer HEPES (KRH) buffer for OCTs and in K^+^-based buffer (KBB) for MATEs as previously described [11,23]. As described previously [18], when applicable, the cells were pre-incubated with Cd^2+^ for 20 min to enhance the activities of transporters, if any. Then, the uptake assay was performed in the presence or absence of Cd^2+^. Cd^2+^ has been previously characterized as a substrate, i.e., competitive inhibitor, toward OCT and MATE transporters by us and others [21,22,23]. The presence of Cd^2+^ in the uptake assays allowed us to examine whether there was any inhibitory effect of Cd^2+^ on transporter activity. The toxicity of Cd^2+^ exposure on HEK cells has been characterized in our previously work [18,23]. A pre-incubation of 20 min and uptake of 20 sec of Cd^2+^ with the concentrations used in the present study did not influence cell viability. For radioactive substrates, radioactivity was measured by a liquid scintillation analyzer (PerkinElmer, Tri-Carb 2910 TR), while cisplatin was quantified by ICP-MS (Agilent 7700). All the uptake data were normalized by the protein level in the lysate.

### 2.8. Animal Studies

Protocol that involved animal studies was approved by the Institutional Animal Care and Use Committee (IACUC) at the University of Maryland, Baltimore (Approved No. 0617011, 10/02/2019). The wild-type mice (C57BL/6J background) and Mate1 knockout mice previously established in our lab [37] were used at 10-16 weeks of age. For in vivo pharmacokinetics studies, the mice were given a single dose of cadmium chloride (2 mg/kg, i.v.) or candesartan (5 mg/kg, i.p.), which was followed by a single dose of metformin (7.5 mg/kg, i.p., with 1/40 [^14^C]-metformin) 60 min after cadmium chloride or candesartan administration. Control mice were given saline or saline with vehicle. The blood samples were collected at 15, 30, 45, 60, 90, and 120 min via tail vein after metformin administration, with a 20 µL of plasma being added into 2 mL of scintillation buffer for radioactivity counting. To determine the tissue accumulation, mice were euthanized 30 min after metformin administration, with liver and kidney being separated, cut, and weighted, and then homogenized in PBS buffer (500 µL). Then, supernatant (300 µL) was added into 3 mL of scintillation buffer for radioactivity quantification.

### 2.9. Statistical Analysis

GraphPad Prism (version 6.01, GraphPad Software, San Diego, CA, USA) was used to perform the statistical analysis. For protein half-life determination, data are expressed as mean ± S.E.M. of three independent experiments. Data were fit by linear regression with half-life being calculated at 50% of degradation. For animal studies, three to four mice in each group were used in all experiments, and the data are expressed mean ± S.E.M. The rest of the data are expressed as mean ± S.D, which is always representative of three independent experiments. A two-tail Student’s t-test was used for statistical comparison between two groups, while one-way analysis of variance (ANOVA) followed by post hoc Turkey test was adopted in comparison among more than two different groups. *p* < 0.05 was considered as the threshold of statistical significance.

## 3. Results

### 3.1. Effects of Cd^2+^ Exposure on the Activities of OCTs and MATEs

**Figure 1:**
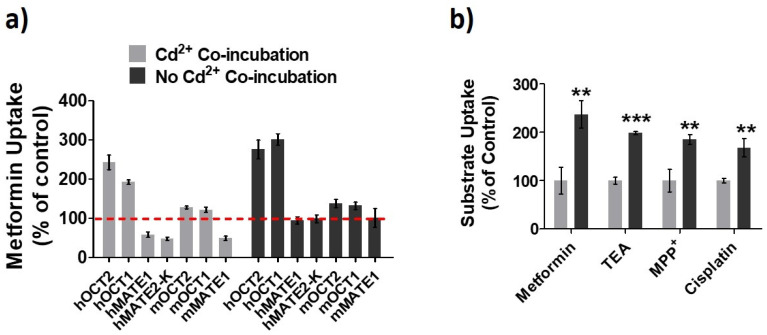
Effects of Cd^2+^ exposure on the activities of organic cation transporters (OCTs) and multidrug and toxin extrusion proteins (MATEs). (**a**) Cellular uptake of metformin in HEK293 cells stably expressing human (h) OCT1, hOCT2, hMATE1, and hMATE2-K; transiently expressing mouse (m) OCT1, mOCT2, and mMATE1 after pre-incubation Cd^2+^ for 20 min. Then, uptake of metformin was performed with (Cd^2+^ Co-incubation) and without Cd^2+^ (No Cd^2+^ Co-incubation). Uptake without Cd^2+^ during both pre-incubation and co-inhibition served as the control. Uptake values are shown as the percentage of control. (**b**) Cellular uptake of metformin, TEA, MPP^+^ and cisplatin in HEK-hOCT2 cells with (dark gray) and without (light gray) Cd^2+^ pre-incubation for 20 min. Data are presented as mean ± S.D.; ** *p* < 0.01, *** *p* < 0.001.

OCTs/MATEs have been implicated in the accumulation and detoxification of Cd^2+^ in renal tubular cells by facilitating its entry and elimination [21,22,23]. Here, we further examined the effects of constant presence of Cd^2+^ on the activities of different OCTs/MATEs for several substrates. The HEK293 cells overexpressing transporter proteins were pre-incubated with Cd^2+^ (100 µM) for 20 min, and the transporter activities were assessed with and without the continuing presence of Cd^2+^ (100 µM). Pre-incubation of cells with Cd^2+^ allowed to observe whether Cd^2+^ could increase the function of the transporters. Meanwhile, because Cd^2+^ is a substrate toward OCTs and MATEs [21,22,23], the co-incubation of cells with Cd^2+^ during the uptake assay allowed examining whether the presence of Cd^2+^ had any inhibitory effect on the activity of the transporters. Our data showed that Cd^2+^ exposure could increase the activities of human (h) OCT1, hOCT2, mouse (m) OCT1, and mOCT2 (Figure 1a). Interestingly, Cd^2+^ behaved as an inhibitor to human and mouse MATEs in the in vitro co-incubation with Cd^2+^. We also verified the increase of hOCT2 activity by Cd^2+^ exposure by testing different substrates, including metformin, TEA, MPP^+^, and cisplatin. A similar magnitude of increase in cellular uptake was observed for these substrates in the cells pre-exposed to Cd^2+^ as compared to the vehicle control (Figure 1b). Taken together, our results indicated that Cd^2+^ could increase the activities of OCTs while behaving as an inhibitor of MATEs.

### 3.2. hOCT2 Was More Active in Cellular Trafficking Than hMATE1

**Figure 2:**
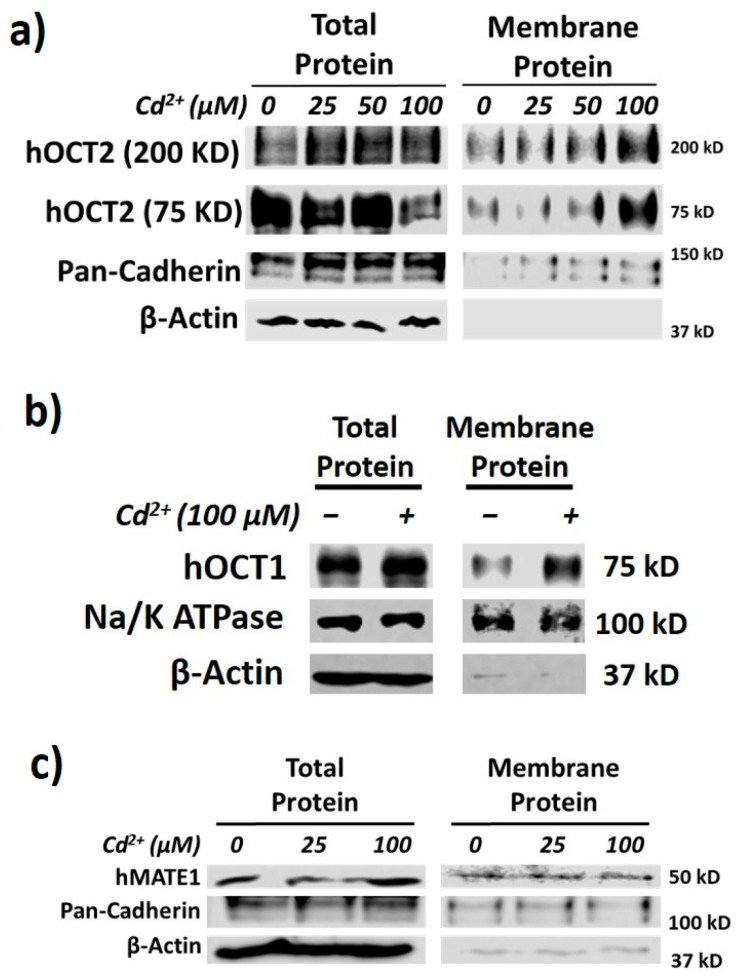
Cd^2+^ exposure increases membrane expression of hOCT1 and hOCT2 but not hMATE1. (**a**) Immunoblotting (IB) analysis of total and membrane fraction of hOCT2 in HEK-hOCT2 cells with and without exposure to different concentrations of Cd^2+^ for 20 min. (**b**) IB analysis of total and membrane fraction of hOCT1 in HEK-hOCT1 cells with and without exposure to Cd^2+^ for 20 min. (**c**) IB analysis of total and membrane fraction of hMATE1 in HEK-hMATE1 cells with and without exposure to different concentrations of Cd^2+^ for 20 min. All figures are representatives of three independent experiments. Pan-cadherin and Na^+^/K^+^ ATPase are served as the positive control and β-actin as negative control for membrane proteins.

As reported recently [18], we firstly confirmed that Cd^2+^ exposure could significantly increase the membrane expression, but not cellular total protein, of hOCT2 (Figure 2a). In fact, we noticed that the total expression of hOCT2 seemed to be lower in the presence of the highest concentration of Cd^2+^ (100 µM). While the overall cell viability was not affected by the experimental conditions, it is likely that the highest concentration of Cd^2+^ might speed up the degradation of hOCT2, the mechanism of which warrants further investigation. As described previously [30], both hOCT2 monomer (≈75 KD) and oligomer (≈200 KD) bands were shown in the Western blot analysis. Interestingly, the surface amount of pan-cadherin was also somewhat increased by Cd^2+^. Pan-cadherin was used as a positive control for membrane protein. The data suggested that the Cd^2+^ effect was not restricted to hOCT2 only, and it might increase the surface amount of some other membrane proteins. Actually, Cd^2+^ exposure could also increase the membrane expression of hOCT1 (Figure 2b). In contrast, Cd^2+^ exposure had no impact on the membrane expression of hMATE1 (Figure 2c). It was likely that the protein trafficking for OCTs and MATEs to the membrane was differentially regulated in response to Cd^2+^ treatment.

**Figure 3:**
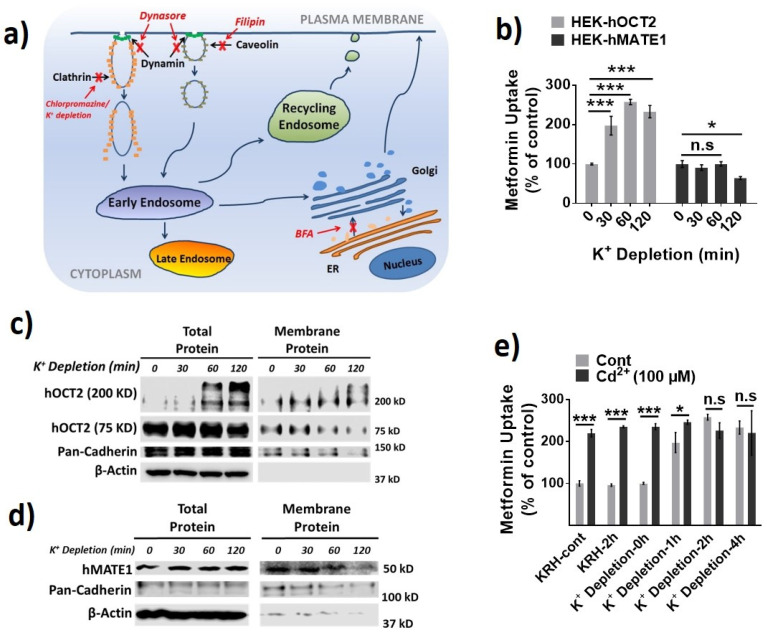
Effect of potassium (K^+^) depletion on the membrane expression and function of hOCT2 and hMATE1. (**a**) A schematic model of the cellular protein trafficking pathways, and the mechanisms of different protein trafficking perpetrators. (**b**) Cellular uptake of metformin in HEK-hOCT2 and HEK-hMATE1 cells after exposure to K^+^ depletion buffer at indicated times. (**c**) IB analysis of total and membrane fraction of hOCT2 in HEK-hOCT2 cells after exposure to K^+^ depletion buffer at indicated times. (**d**) IB analysis of total and membrane fraction of hMATE1 in HEK-hMATE1 cells after exposure to K^+^ depletion buffer at indicated times. (**e**) Cellular uptake of metformin in HEK-hOCT2 cells with and without Cd^2+^ treatment for 20 min after being exposed to K^+^ depletion buffer at indicated times. For c and d, pan-cadherin served as the positive control and β-actin as the negative control for membrane proteins. For b and e, data are presented as mean ± S.D.; * *p* < 0.05, *** *p* < 0.001.

**Figure 4:**
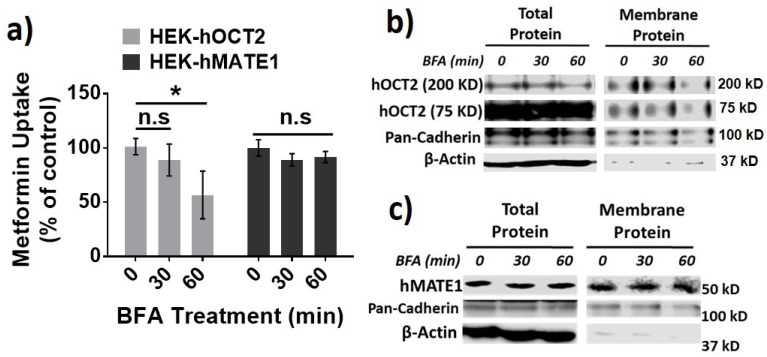
Effect of brefeldin (BFA) treatment on the membrane expression and function of hOCT2 and hMATE1. (**a**) Cellular uptake of metformin in HEK-hOCT2 and HEK-hMATE1 cells after exposure to BFA at indicated times. (**b**) IB analysis of total and membrane fraction of hOCT2 in HEK-hOCT2 cells after exposure to BFA at indicated times. (**c**) IB analysis of total and membrane fraction of hMATE1 in HEK-hMATE1 cells after exposure to BFA at indicated times. For b and c, pan-cadherin is served as the positive control and β-actin as the negative control for membrane proteins. For a, data are presented as mean ± S.D.; * *p* < 0.05; n.s., no significance.

**Figure 5:**
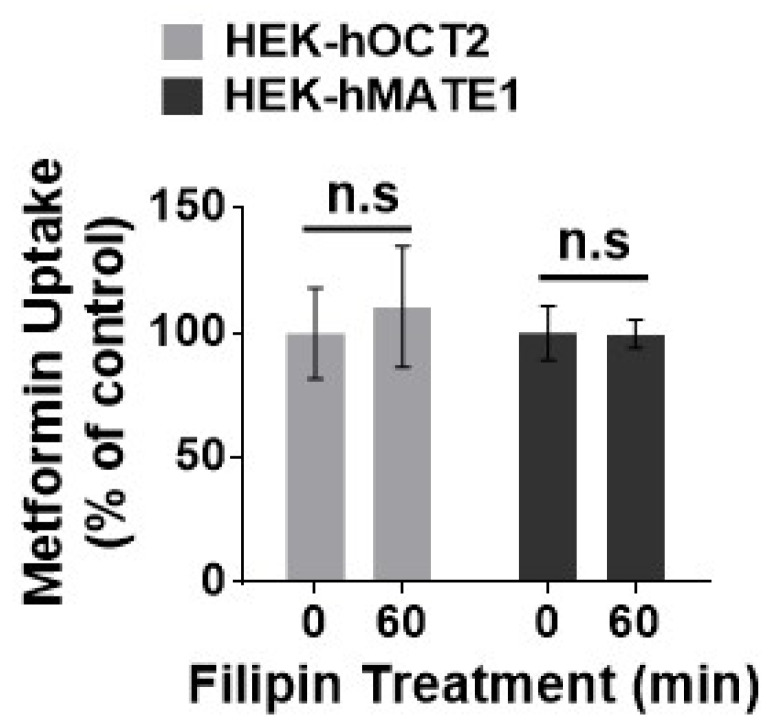
Effect of filipin on the function of hOCT2 and hMATE1. Cellular uptake of metformin in HEK-hOCT2 and HEK-hMATE1 cells after being exposed to filipin at indicated times. Data are presented as mean ± S.D.; n.s., no significance.

Then, we investigated the role of different protein trafficking pathways in the membrane expression and activity for hOCT2 and hMATE1, by using several well-known perpetrators of protein trafficking, including potassium (K^+^) depletion [38], brefeldin A (BFA) [39], and filipin [40] (Figure 3a). Potassium depletion, a selective blocker of the clathrin-dependent endocytosis, could time-dependently increase the activity of hOCT2 as determined from metformin uptake (Figure 3b), and the abundance of hOCT2 oligomers (Figure 3c). In contrast, potassium depletion up to 60 min had little effect on the activity and membrane protein expression of hMATE1 (Figure 3b,d). In particular, we found that the increased hOCT2-mediated metformin uptake by Cd^2+^ could be abolished by potassium depletion (Figure 3e), which is probably due to the depletion of cytosol hOCT2 pool. Likewise, the treatment of BFA, a chemical that can inhibit protein transport from the endoplasmic reticulum (ER) to the Golgi apparatus, could significantly reduce the activity and membrane protein expression of hOCT2 (Figure 4a,b), but it showed no impact on hMATE1 membrane expression (Figure 4a,c). However, the blocking of caveolin-dependent endocytosis by filipin had no influence on the activity of both hOCT2 and hMATE1 (Figure 5).

**Figure 6:**
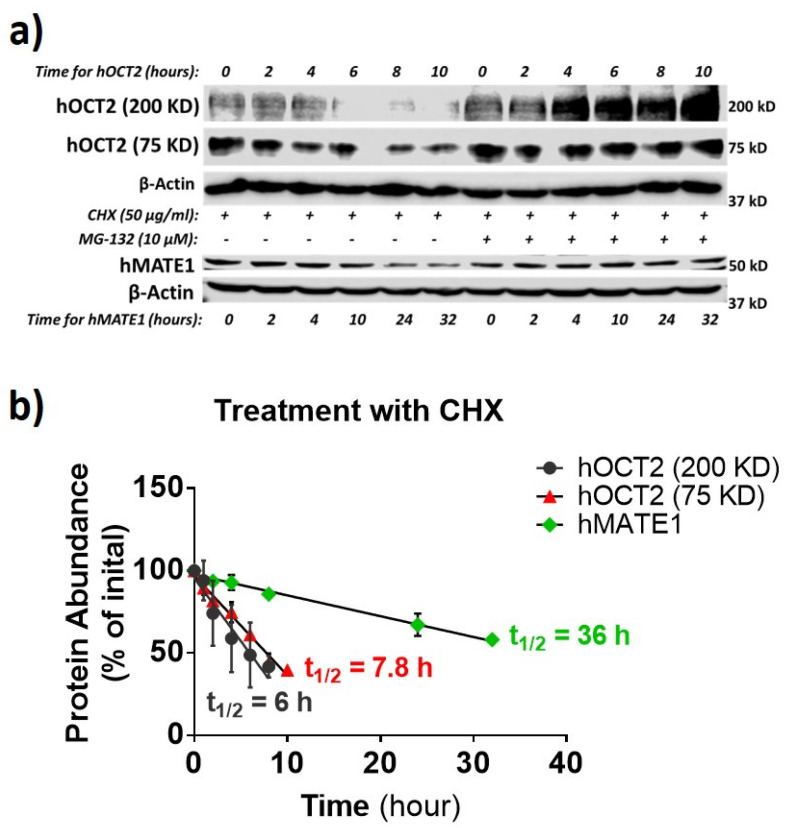
hMATE1 has a much longer half-life than hOCT2. (**a**) IB analysis of hOCT2 and hMATE1 after being exposed to translation inhibitor cycloheximide (CHX) or CHX plus MG-132 (proteasome inhibitor) at indicated times. β-actin is used as the loading control. (**b**) Calculation of the protein degradation rate as half-life (t1⁄2), by fitting the data with a linear regression mode. Half-lives of hOCT2 and hMATE1 were determined as the time of 50% degradation achieved under CHX treatment.

The degradation half-life of a membrane protein may reflect how active it is synthesized and in the trafficking between the cytosol pool and the membrane. Thus, the protein half-life for hOCT2 and hMATE1 was examined by using the protein translation inhibitor cycloheximide (CHX) and proteasome inhibitor MG-132 (Figure 6a,b), as described previously [41,42]. The oligomer and the monomer of hOCT2 exhibited a similar half-life (≈6 h and ≈8 h respectively), which was much shorter than that of hMATE1 (≈36 h).

Together, our findings suggested that hOCT2 was a short-lived protein under active trafficking between plasma membrane and cytoplasmic storage pool, and hence, it was susceptible to the translocation regulation by Cd^2+^ exposure. On the contrary, hMATE1 was a more static and long-lived protein in the cell membrane, being less susceptible to such a regulation by Cd^2+^ exposure.

### 3.3. Cd^2+^ Exposure Altered the Pharmacokinetics (PK) of Metformin in Mice

**Figure 7:**
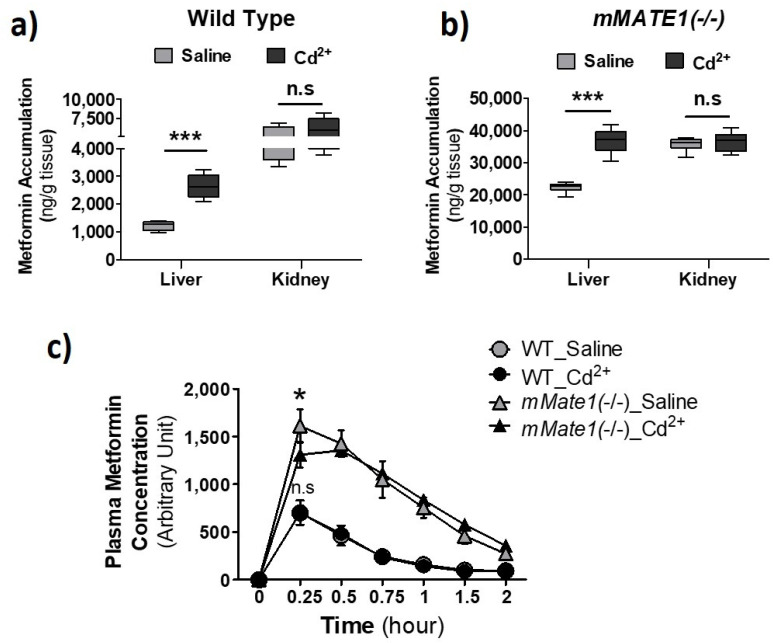
Effect of Cd^2+^ exposure on the pharmacokinetics (PK) of metformin in mice. (**a**,**b**) Accumulation of metformin in liver and kidney after wild-type (*WT*) and *mMATE1* knockout(−/−) mice being treated with and without CdCl_2_ (2 mg/kg, i.v.) for 1 h, which was followed by a single dose of metformin (7.5 mg/kg, i.p.). Tissues were collected 30 min after metformin administration. (**c**) Quantification of plasma level of metformin in *WT* and *mMATE1*(−/−) mice after being treated with and without CdCl_2_ (2 mg/kg, i.v.) for 1 h, which was followed by a single dose of metformin (7.5 mg/kg, i.p.). Blood samples were collected from the tail vein at 0.25, 0.5, 0.75, 1.0, and 2.0 h after metformin administration. For (**a**,**b**), *n* = 4; for c, *n* = 3 or 4. Data are presented as mean ± S.E.M.; * *p* < 0.05, *** *p* < 0.001; n.s., no significance.

To further obtain the clinical implication of our findings, we examined the effect of Cd^2+^ exposure on the pharmacokinetics (PK) of metformin in wild-type (Wt) and Mate1 knockout (−/−) mice. Mate1(−/−) mice would enable us to determine the effect resulting from the alteration of Oct activity alone, while the effect via both Oct and Mate activities would be observed in Wt mice. Our data showed that Cd^2+^ exposure could increase the accumulation of metformin by nearly 2.5 fold in the liver of Wt mice, as compared with 1.5-fold increase in Mate1(−/−) mice, supporting a combined effect of Oct activity increase and Mate inhibition by Cd^2+^ exposure in Wt mice (Figure 7a,b). The accumulation of metformin was unchanged by Cd^2+^ exposure in the kidney of both Wt and Mate1(−/−) mice. Interestingly, we found that Cd^2+^ exposure could significantly decrease the maximal plasma concentration of metformin (at 0.25 h) after metformin administration in Mate1(−/−) mice, while having no impact on systemic metformin exposure in Wt mice (Figure 7c). Collectively, our results demonstrated that Cd^2+^ exposure could alter the PK of metformin in vivo probably through its effects on Oct and Mate transporter activities.

### 3.4. Increase of OCT2 Activity by Candesartan

**Figure 8:**
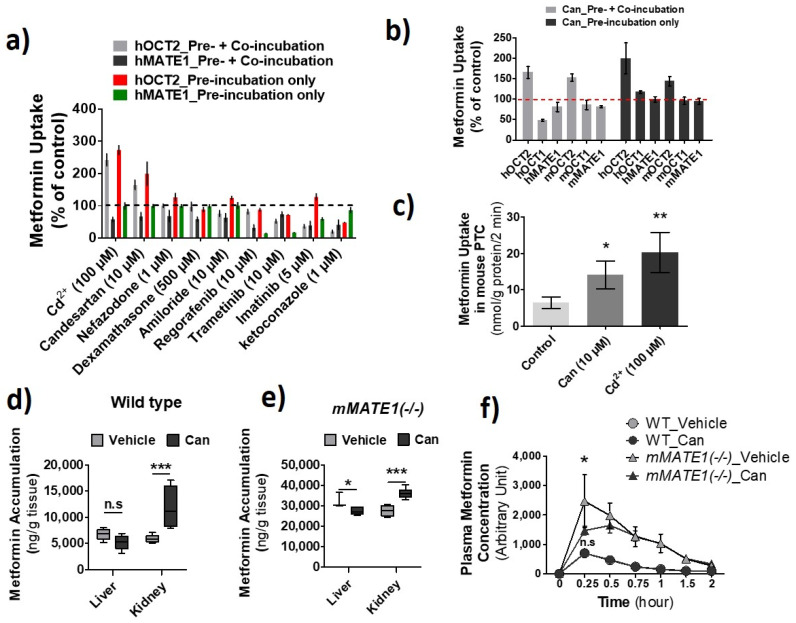
Identification of candesartan (Can) as an agent increasing the activity of OCT2 and inhibiting the activity of multidrug and toxin extrusion proteins (MATEs). (**a**) Cellular uptake of metformin in HEK-hOCT2 and HEK-hMATE1 cells after exposure to different drugs at indicated concentrations for 30 min. (**b**) Cellular uptake of metformin in HEK293 cells stably expressing human (h) OCT1, hOCT2, hMATE1, and hMATE2-K; transiently expressing mOCT1, mOCT2, and mMATE1 after exposure to candesartan for 30 min. (**a**,**b**) Then, the uptake of metformin was performed with (Pre-incubation + Co-incubation) and without different drugs (Pre-incubation only). Uptake without drugs during pre-incubation and co-inhibition served as the control. Uptake values are shown as percentage of control. (**c**) Cellular uptake of metformin in mouse primary renal tubular cells after exposure to Cd^2+^ and candesartan at the indicated concentrations for 20 min. (**d**,**e**) Accumulation of metformin in the liver and kidney after wild-type (*Wt*) and *mMATE1(−/−)* mice being treated with and without candesartan (can) (5 mg/kg, i.p.) for 1 h, which was followed by a single dose of metformin (7.5 mg/kg, i.p.). Tissues were collected 30 min after metformin administration. (**f**) Quantification of plasma levels of metformin in WT and *mMATE1(−/−)* mice after being treated with and without candesartan (Can) (5 mg/kg, i.p.) for 1 h, then a single dose of metformin (7.5 mg/kg, i.p.). Blood samples were collected from tail vein at 0.25, 0.5, 0.75, 1.0, and 2.0 h after metformin administration. Uptake values are shown as percentage of control. For d and e, n = 4; for f, n = 3 or 4. * *p* < 0.05, ** *p* < 0.01, *** *p* < 0.001; n.s., no significance.

Previously, we have examined a collection of clinically used drugs for their inhibitory potency against hOCT2, hMATE1, and hMATE2-K [26]. For the same set of drugs, we continued testing whether those non-hOCT2 inhibitors could increase the activity of hOCT2 instead. We identified candesartan as such a drug (Figure 8a). At 10 µM, candesartan pre-incubation significantly increased metformin uptake mediated by hOCT2 and mOct2 in HEK293 cells but inhibited the uptake by hMATE1 and mMTE1 under co-incubation conditions. Interestingly, candesartan was also an inhibitor toward hOCT1 but with little inhibition on mOCT1 at the tested concentration (Figure 8b). We further examined the effect of candesartan and Cd^2+^ on metformin uptake in mouse primary renal tubular cells, from which a two- and three-fold increase of accumulation was observed, respectively (Figure 8c). Then, we examined the effect of this relatively specific enhancer for OCT2 activity on the PK of metformin in Wt and Mate1(−/−) mice. Consistent with the results in vitro, candesartan (5 mg/kg, i.p.) could significantly increase renal metformin accumulation in both Wt and Mate1(−/−) mice, while it could slightly decrease the accumulation in the liver. A nearly 2-fold increase was seen in the kidney of Wt mice; in contrast, only 30% increase was observed in the kidney of Mate1(−/−) mice (Figure 8d,e). Similar to the results by Cd^2+^ above, we found that candesartan could significantly decrease the maximal plasma metformin level in Mate1(−/−) mice after metformin injection while having no impact on metformin levels in Wt mouse plasma (Figure 8f). These results further proved the concept that OCT activity increase and/or MATE inhibition may lead to alteration in drug disposition.

## 4. Discussion

The understanding of regulation of OCTs/MATEs remains lagged far behind the extensive research demonstrating the critical role of these transporters in drug disposition. In this study, we have characterized intriguing divergent effects of Cd^2+^ exposure in regulation of the activities of OCTs and MATEs. Specifically, we have shown that hOCT2 protein, not hMATE1, was actively trafficking between plasma membrane and cytoplasmic storage pool, which was subject to the regulation by Cd^2+^ exposure. Cd^2+^ exposure increased the activities of OCTs but served as an inhibitor of MATE transporters. In addition, we identified that candesartan, a widely prescribed drug, could specifically increase OCT2 activity and inhibit MATE activities. With either Cd^2+^ or candesartan, we have proved the concept that the increase of OCT activities along with the inhibition of MATEs could significantly enhance the accumulation of the substrates of these transporters in the liver and/or kidney. Overall, our results have demonstrated that certain xenobiotics may affect drug disposition via their stimulatory effect on OCT membrane translocation as well as their inhibition towards MATEs.

The divergent effects of Cd^2+^ in the regulation of OCTs and MATEs were likely related to the dynamic differences in protein trafficking between the two transporter families. We found that hOCT2 was a relatively short-lived protein under actively trafficking, while hMATE1 had a much longer half-life as a more static protein in the cell membrane. Our findings are consistent with previous reports in which the regulation of protein trafficking has been linked to the changes in the function and/or membrane expression of OCT2 [13,16,18,43]. However, there has been no reports showing that MATE proteins are susceptible to any regulators of protein trafficking. It is unclear why the cells preserve such an instant adaptation mechanism for OCTs but not for MATEs. Notably, we only employed chemical regulators to characterize the role of protein trafficking pathways in the membrane expression and activity of these transporters. These chemical regulators may not be specific and could inevitably bring in off-target effects. For example, we found that dynasore and chlorpromazine, the inhibitors of dynamin and clathrin-mediated endocytosis respectively [44,45], were also potent inhibitors of OCTs (data not shown), making them inappropriate tools to study the role of dynamin and clathrin-dependent endocytosis in the regulation of OCTs. Future studies with a genetic approach to specifically knock down those key regulators of protein trafficking, including clathrin, caveolin, and dynamin, should be used to validate our findings in the present study.

It has to be mentioned that we observed a weaker effect of both Cd^2+^ and candesartan on mOCT2 as compared to hOCT2 in HEK cells; however, a similar effect could be seen in mouse primary renal tubular cells (Figure 1a and Figure 8b,c). Since a transient transfection was used for the expression of mOCT2 as compared to the stable expression for hOCT2, we assumed that the transient transfection, in which a large amount of proteins were synthesized in a short period of time, might significantly interfere with the process of protein trafficking, leading to the weaker stimulatory effect observed. Moreover, while our findings were extended from our recent work [18] and validated with mouse primary renal tubular cells and animals, it should be noted that they were mainly obtained with the cells artificially overexpressing the transporters. In addition, although we have examined the effect of Cd^2+^ and candesartan on the activities of major human and mouse OCTs and MATEs, the effect may be different on other OCT and MATE homologs.

We did not observe an increase of metformin accumulation in mouse kidney in comparison to the liver when CdCl_2_ was acutely injected. We reasoned that this was due to the unique toxicokinetics of inorganic Cd^2+^ which is firstly distributed in the liver [46,47,48,49] where Cd^2+^ stimulates the expression of, and is bound to, proteins such as metallothionein (MT). Then, the Cd^2+^ bound to MT (CdMT) and other proteins is re-distributed, mainly into the kidney. We have confirmed that the Cd^2+^ level in the kidney was much lower than that in the liver after acute CdCl_2_ administration in mice (data not shown). On the other hand, we did not observe an increase of metformin accumlation in mouse liver in comparison to the kidney when a single dose of candesartan was administrated. This might be, at least partially due to the fact that candesartan had a relatively specific effect on the activity OCT2, including mOCT2, toward which the probe drug metformin is a preferred substrate [50]. mOCT1 is a relatively specific transporter in the liver, while both mOCT1 and mOCT2 are highly expressed in the kidney [51]. It is unknown why candesartan had a specific effect on the activity of OCT2 over OCT1. In the future, it will be interesting to characterize the mechanism underlying the effect of candesartan on OCT2 activity and compare it with that by Cd^2+^.

It is important to know that Cd^2+^ and candesartan did not increase the activity of MATE transporters, which share a wide spectrum of substrates and collaborate with OCTs for transcellular drug transport in the kidney and liver. Instead, because Cd^2+^ is a substrate of MATEs [23], a high concentration of intracellular Cd^2+^ is expect to competitively inhibit MATE-mediated drug efflux. Candesartan has also been characterized as a moderate inhibitor of MATEs previously [26]. Consistent with the dual effects of OCT activity increase and MATE inhibition by Cd^2+^ and candesartan, we observed that the magnitude of increase in metformin accumulation in the liver or kidney after Cd^2+^ or candesartan treatment was less in *Mate1(−/−)* mice as compared to that in *Wt* mice. Interestingly, while Cd^2+^ and candesartan could increase metformin accumulation in the liver and kidney respectively, they had little impact on the systemic exposure of metformin in mice, particularly in *Wt* mice. We postulated that it was probably because the effects of OCT activity increase and MATE inhibition by Cd^2+^ and candesartan allowed the substrates such as metformin to remain in the liver and/or kidney tissues and cancelled out the impact of each other on the systemic exposure of their substrates. Our data suggested that a drug–drug interaction (DDI) causing significant drug accumulation in tissues may not necessarily lead to a significant change in the systemic exposure of the affected drug.

Collectively, we have found that the activities of OCTs and MATEs could be divergently affected by Cd^2+^ or candesartan treatment, which was probably due to the distinct dynamics of protein trafficking between the two transporter families. The dual effects of OCT activity increase and MATE activity inhibition by certain xenobiotics such as Cd^2+^ and candesartan may lead to significant accumulation of the substrate compounds of these transporters in the liver or kidney. Future studies are needed to understand the clinical implication of these complex OCT/MATE-based DDIs.

## Data Availability

Not applicable.
